# Characterization of hepatic macrophages and evaluation of inflammatory response in heme oxygenase-1 deficient mice exposed to scAAV9 vectors

**DOI:** 10.1371/journal.pone.0240691

**Published:** 2020-10-15

**Authors:** Mateusz Tomczyk, Izabela Kraszewska, Robert Mąka, Agnieszka Waligórska, Józef Dulak, Agnieszka Jaźwa-Kusior

**Affiliations:** 1 Department of Medical Biotechnology, Faculty of Biochemistry, Biophysics and Biotechnology, Jagiellonian University, Kraków, Poland; 2 Department of Cell Biophysics, Faculty of Biochemistry, Biophysics and Biotechnology, Jagiellonian University, Kraków, Poland; University of Florida, UNITED STATES

## Abstract

Adeno-associated viral (AAV) vectors are characterised by low immunogenicity, although humoral and cellular responses may be triggered upon infection. Following systemic administration high levels of vector particles accumulate within the liver. Kupffer cells (KCs) are liver resident macrophages and an important part of the liver innate immune system. Decreased functional activity of KCs can contribute to exaggerated inflammatory response upon antigen exposure. Heme oxygenase-1 (HO-1) deficiency is associated with considerably reduced numbers of KCs. In this study we aimed to investigate the inflammatory responses in liver and to characterise two populations of hepatic macrophages in adult wild type (WT) and HO-1 knockout (KO) mice following systemic administration of one or two doses (separated by 3 months) of self-complementary (sc)AAV9 vectors. At steady state, the livers of HO-1 KO mice contained significantly higher numbers of monocyte-derived macrophages (MDMs), but significantly less KCs than their WT littermates. Three days after re-administration of scAAV9 we observed increased mRNA level of monocyte chemoattractant protein-1 (*Mcp-1*) in the livers of both WT and HO-1 KO mice, but the protein level and the macrophage infiltration were not affected. Three days after the 1^st^ and 3 days after the 2^nd^ vector dose the numbers of AAV genomes in the liver were comparable between both genotypes indicating similar transduction efficiency, but the percentage of transgene-expressing MDMs and KCs was higher in WT than in HO-1 KO mice. In the primary culture, KCs were able to internalize AAV9 particles without induction of TLR9-mediated immune responses, but no transgene expression was observed. In conclusion, *in vivo* and *in vitro* cultured KCs have different susceptibility to scAAV9 vectors. Regardless of the presence or absence of HO-1 and initial numbers of KCs in the liver, scAAV9 exhibits a low potential to stimulate inflammatory response at the analysed time points.

## Introduction

Recombinant adeno-associated viral (AAV) vectors, derived from a replication-defective, non-pathogenic parvovirus with a small single-stranded (ss) DNA genome, have emerged as an attractive gene therapy tool due to safe, stable and long-term transgene expression. The genome of the recombinant AAV vectors, in contrary to the genome of the wild type virus, does not undergo site-specific integration into the host DNA but mainly remains in the episomal form in the nuclei of transduced cells [1]. Following cellular entry and translocation to the nucleus, ssAAV genome must be converted to a double-stranded (ds) form to enable transgene expression. This rate-limiting step in AAV transduction is omitted when the self-complementary (sc) AAV vectors, developed by the elimination of a nicking site in one of the inverted terminal repeats (ITRs) [2], are used. AAV vectors generated from several naturally occurring AAV serotypes have different cell tropism and kinetics of transgene expression (reviewed in: [1]). Their ability to efficiently transduce terminally differentiated or non-proliferating cells provides considerable, long-term *in vivo* gene transfer in various cell types e.g. muscle cells, hepatocytes, neurons, retinal cells, and many others (reviewed in: [1]). Several AAV serotypes are capable of body-wide transduction after a single intravascular injection [3–5], even though in certain diseases, utilisation of more selective serotypes might be beneficial. For example, in patients suffering from Duchenne muscular dystrophy the application of AAV serotype 9 (AAV9), which enables high and long-term transgene expression in the heart and skeletal muscles [5, 6], may be advantageous. At the same time, however, systemic delivery of AAV9 results in liver transduction [5].

Apart from its crucial role in metabolism, liver plays an important role in the systemic immune response [7]. The organ relies on its own immune system consisting of macrophages, natural killer cells and T cells [8], to protect itself from damage due to different pathogens and toxic agents that enter the liver *via* the portal vein or hepatic artery. On the other hand, this uniquely specialised immune system of the liver, conferring the so-called immunotolerance, ensures that the liver does not mount a strong immune response against harmless self, dietary, and commensal organism antigens that arrive from the circulation *via* the hepatic artery and from the gastrointestinal tract *via* the portal vein. Hepatic macrophages are defined as a heterogeneous population of immune cells derived either from circulating monocytes or from fetal yolk sac and local precursors [9]. The first population, in mice called Ly6C^hi^ monocyte-derived macrophages (MDMs), is massively recruited from the bloodstream to the injured liver and is linked to chronic inflammation and fibrosis [9]. The latter population consists of Kupffer cells (KCs) and is maintained through self-renewing divisions [10, 11]. Importantly, recent studies demonstrate that following depletion of liver-resident KCs, circulating monocytes engraft in the emptied niches of the liver and gradually become long-lived self-renewing cells [12, 13]. KCs are predominantly localized in the hepatic sinusoids what enables a direct contact with blood-derived leukocytes and facilitates the clearance of pathogens entering the liver with blood. As such, KCs have been implicated in both immunogenic and tolerogenic immune reactions [14–16].

AAV vectors can activate innate immune response due to interaction with Toll-like receptors (TLRs), either through capsid or viral genome recognition by TLR-2 and TLR-9, respectively. This leads to NFκB‐mediated upregulation of proinflammatory cytokines such as tumor necrosis factor α (TNFα), interleukin (IL)-6, IL‐8 and several components of the IL‐1 pathway [17–19]. However, it has been shown that activation of such response *in vivo* is transient, self-limited and resolved within several hours after vector administration [17, 20]. In general, AAV vectors are characterised by low immunogenicity, although humoral as well as T cell responses in patients may be induced [21, 22]. Exposure to wild-type AAV or systemic administration of the AAV vector leads to the generation of circulating antibodies directed against the AAV capsid [21, 22]. Binding of neutralizing antibodies (nAbs), targeting viral epitopes critical for cellular entry or vector disassembly, to the capsid limits infectivity of the viral vector and may have a profound effect on gene transfer efficiency [23, 24]. The use of alternate capsids, immune suppression and decoy capsid approaches partially solved this serious problem in the gene therapy field [25]. As susceptibility of AAV vectors to nAbs can vary depending on various, yet not completely understood factors, the presence of pre-existing antibodies may not necessarily exclude subjects from a particular gene therapy. For example a recent study by Majowicz *et al* shows that hemophilia B patients can be successfully treated by systemic administration of AAV5-based vectors in the presence of detectable pre-existing anti-AAV5 nAbs [26].

Heme oxygenase-1 (HO-1, *HMOX1*) catalyzes conversion of heme to biliverdin, carbon monoxide (CO), and ferrous iron (Fe^2+^). Through the first two compounds and upregulation of ferritin by iron ions, HO-1 exerts cytoprotective and immunomodulatory effects [27]. Indeed, this protein has been shown to regulate both innate and adaptive immune responses [28]. Moreover, HO-1 knockout (KO) mice were previously characterized by enhanced liver inflammation and considerably reduced numbers of KCs due to toxicity of the accumulated heme following phagocytosis of senescent red blood cells [29]. It was also shown that liver macrophages in HO-1 KO mice present a pro-inflammatory and activated phenotype [30]. So far, there have only been two human cases of HO-1 deficiency described [31, 32], but the magnitude of HO-1 expression may be influenced by polymorphisms in the *HMOX1* gene promoter [33, 34]. Increased monocytic *HMOX1* expression was associated with reduced markers of inflammatory polarization [35].

The aim of this study was to investigate the inflammatory responses in liver and to characterise two populations of hepatic macrophages following systemic injection of scAAV9 vectors in wild type (WT) and HO-1 KO mice, the latter characterised by significantly reduced Kupffer cell numbers. To promote the *in vivo* response to AAVs, we administered intravenously two doses (separated by 3 months) of either an empty vector (without transgene-coding sequence) or a vector carrying a reporter gene—green fluorescent protein (GFP). The classical animal models to study the impact of pre-existing immunity are based on vector re-administration 4 weeks after the first injection [36]. However, administration of the 2^nd^ dose of vector 3 months after the 1^st^ one, should be more clinically relevant. In this case, very high anti-AAV antibody titers, which presumably would only be seen in humans after a recent exposure [21], can be avoided. On the 3^rd^ day after delivery of the second dose of scAAV9 we observed increased monocyte chemoattractant protein-1 (*Mcp-1*) mRNA level in the livers of both WT and HO-1 KO mice, but the protein level and the macrophage infiltration were not affected. Plasma samples collected 3 days after vector re-administration demonstrated considerable and significantly higher in HO-1 KO than in WT mice *in vitro* neutralizing activity to AAV9. In addition, a robust transgene expression in the two analyzed populations of hepatic macrophages was observed with the higher numbers of transgene-expressing cells in WT than in HO-1 KO mice. In the primary culture, KCs were able to internalize AAV9 without induction of TLR9-mediated immune responses. However, in contrast to the animal studies, no transgene expression was observed in the cultured KCs.

## Materials and methods

### AAV production

Production of scAAV9 vectors was performed using CellRoll Bottle Roller (Integra) in three-plasmid Helper-free system. Briefly, HEK293 cells were seeded on collagen-coated polystyrene Roller Bottles (Corning), cultured until they reached 50–60% confluence and co-transfected with 130 μg of pHelper, 100 μg of p5E18VD2/9 and 90 μg of pdAAV-CMV-Empty (containing CMV promoter, but devoid of transgene-coding sequence) or pdAAV-CMV-GFP plasmids, using polyethylenimine (linear, MW 25000, Polysciences) as a transfection reagent (2.58 mg/ml; 1 μl per 1 μg of DNA). After 72 hours, cells were detached from the culture surface, centrifuged (10 min, 350×*g*), washed in PBS and resuspended in a small volume of PBS containing calcium and magnesium. Then, the cells were lysed by 3 cycles of freezing in liquid nitrogen and thawing in 37°C water bath with vigorous mixing after each cycle. The lysate was digested with HS nuclease (MoBiTec) in final concentration of 50 U/ml for 1 hour at 37°C. Next, lysates were cleared twice through centrifugation, each for 30 min at 4000×*g* at 4°C and subsequently subjected to purification by ultracentrifugation on discontinuous iodixanol density gradient. The gradient was built of processed crude lysate placed on 15%, 25%, 40% and 54% iodixanol layers. Iodixanol dilutions were prepared in PBS with 0.5 mM MgCl_2_ and 2.5 mM KCl. After 2 hours of centrifugation in angular rotor at 300 000×*g*, 18°C, AAV-containing fraction (40%) was collected with a needle and syringe and subjected to concentration procedure on Amicon® Ultra-15 Centifugal Filters (100 kDa, Merck Millipore). Quantitative PCR-based titration of AAV genome copies in isolated DNA was performed with ITR-binding TaqMan probe and TaqMan Gene Expression Master Mix, according to manufacturer’s instructions. Final concentration of specific primers and TaqMan probe equalled to 100 nM (For: 5’-CGG CCT CAG TGA GCG A-3; Rev: 5’-GGA ACC CCT AGT GAT GGA GTT-3’; probe: 5’-6-FAM-CAC TCC CTC TCT GCG CGC TCG-BHQ-1-3’).

### Animal procedures

All animal procedures were in accordance with *Guide for the Care and Use of Laboratory Animals* (Directive 2010/63/EU of European Parliament) and carried out under a license from the Ethical Committee of the Jagiellonian University (approval number: 64/2013). In all the experiments we used 3-4-months-old females of C57BL/6×FVB background, wild type (WT) or heme oxygenase-1 knock-out (HO-1 KO) specific pathogen free (SPF) mice. Mice had access to food and water *ad libitum*. Before AAV administration, mice were anesthetized with 5% isoflurane. During AAV injection isoflurane was reduced to 1.5–2%. The limb withdrawal response to toe pinch was monitored to ensure the adequacy of anesthesia. In all, 5×10^11^ vector genome (vg) equivalents of scAAV9 vectors were injected into the jugular vein of WT and HO-1 KO mice as 150 μl bolus using a sterile syringe and 29G needle. Three days post-1^st^ injection, part of the animals was euthanized and peripheral blood and liver samples were collected for further analysis. Three months after the 1^st^ vector administration another group of mice received the 2^nd^ dose of scAAV9 (1×10^11^ vg/mouse). Three days after the 2^nd^ vector dose the animals were euthanized and peripheral blood and liver samples were collected for further analysis.

In the end of the experiment, mice were injected intraperitoneally with a mixture of ketamine hydrochloride (200 mg/kg) and xylazine hydrochloride (40 mg/kg). Then, blood was collected by cardiac puncture using a 22G needle and syringe containing heparin as an anticoagulant (final concentration: 10 U/ml of blood). Subsequently, the liver was perfused *in situ* with 5 ml PBS containing 0.5 U/ml heparin *via* left ventricle punctured with PE-50 catheter connected to a perfusion apparatus (3 ml/minute flow rate). Each liver tissue was divided into several pieces used directly in flow cytometry or stored frozen in −80°C for RNA, DNA or protein analysis. For histological analyses, one piece of each tissue was embedded in paraffin and another one in tissue freezing medium. Blood samples were centrifuged (800×*g*, 10 min, 4°C) and the collected plasma was transferred to new tube and stored frozen at −80°C.

### Immunophenotyping of liver macrophages using flow cytometry

For preparation of a single cell suspension liver was excised, placed in saline on ice, finely minced with surgical scissors and finally incubated in a mixture of 5 mg/ml of collagenase type II (Gibco) and 1.2 U/ml of dispase (Gibco) in PBS with calcium and magnesium ions (Lonza) for 1 hour at 37°C with gentle agitation. Then, equal volume of Dulbecco’s Modified Eagle’s Medium with 4.5 g/l glucose (DMEM HG; Lonza) supplemented with 10% Fetal Bovine Serum (FBS; Gibco) was added. Dissociated cells and undigested tissue were centrifuged at 200×*g* for 5 minutes and the pellet was resuspended in 5 ml of PBS (Lonza). Cell/tissue suspension was passed through 40 μm filters, centrifuged and resuspended in PBS until further analysis. Before staining, the single cell suspension was incubated for 10 minutes on ice with Fc receptor Blocking Reagent (Miltenyi Biotec) in PBS supplemented with 2% FBS. Then, the cells were incubated for 30 minutes at 4°C with the following antibody mix: BV605-conjugated rat anti-mouse CD45 (clone: 30-F11; BD Biosciences), AF700-conjugated rat anti-mouse CD11c (Clone: N418, BD Biosciences); APC-Cy7-conjugated rat anti-mouse CD11b (Clone: M1/70, BD Biosciences) and APC-conjugated rat anti-mouse F4/80 (Clone: BM8, BD Biosciences). DAPI was used for the exclusion of nonviable cells. The cells were washed with PBS and analyzed using LSRFortessa cytometer (BD Biosciences) and FacsDiva (BD Biosciences) or FlowJo 10 (FlowJo, LLC) Software. Unstained, single stained, and fluorescence-minus-one (FMO) controls were used for setting compensation and gates.

### RNA isolation and quantitative PCR

Liver fragments were collected, snap-frozen in liquid nitrogen and stored at −80°C. Total RNA was isolated through modified phenol-chloroform extraction, followed by isopropanol precipitation. Lysis of the tissue was performed using Tissue Lyzer (Qiagen) in 1 ml of Qiazol (Qiagen) reagent. In case of *in vitro* cultured Kupffer cells, RNA isolation was carried out using Single Cell RNA purification Kit (NORGEN). Concentration and quality of the RNA was determined through absorbance measurement at 230, 260 and 280 nm utilizing NanoDrop1000 Spectrophotometer (Thermo Fisher Scientific). Synthesis of cDNA was performed with RevertAid reverse transcriptase (Thermo Fisher Scientific) from 1 μg of liver RNA or with High-Capacity cDNA Reverse Transcription Kit (Applied Biosystems) from KCs RNA. Obtained cDNA was diluted to the final concentration of 10 ng/μl in nuclease-free water. Quantitative PCR (qPCR) was performed using StepOne Plus Real-Time PCR (Applied Biosystems) with reaction mix composed of SYBR Green PCR Master Mix (SYBR^®^ Green JumpStart™ *Taq*, Sigma), 20 ng of a template and specific primers (final 500 nM; Table 1). Elongation factor 2 (*Eef2*) served as a housekeeping gene in the mRNA analyses. Results and product melt curves were analysed using software provided by the manufacturer.

**Table 1:** List of specific primer pairs.

Target	Primer sequence	T_a_ [^o^C]
*Eef2*	For: 5’-AAAAGTATGAGTGGGACGTTGC-3’; Rev: 5’-CCTTGATCTCATTCAGGTACTGC-3’	60
*Mcp1*	For: 5’-CCCAATGAGTAGGCTGGAGA-3’; Rev: 5’-TCTGGACCCATTCCTTCTTG-3’	58
*Tnfa*	For: 5’-TACTGAACTTCGGGGTGATCGGTCC-3’; Rev: 5’-CAGCCTTGTCCCTTGAAGAGAACC-3’	61
*Il10*	For: 5’-AAGGGTTACTTGGGTTGCCA-3’; Rev: 5’-GAGAAATCGATGACAGCGCC-3’	60
*Tlr9*	For: 5’-CCAACCTGCGGCAGCTGAAC-3’; Rev: 5’-TGGGCTCAATGGTCATGTGGCA-3’	60
*Myd88*	For: 5’-CGCAGTTTGTTGGATGCCTG-3’; Rev: 5’-TCGAAAAGTTCCGGCGTTTG-3’	60
*Ifna*	For: 5’-GGACTTTGGATTCCCGCAGGAGAAG-3’; Rev: 5’-GCTGCATCAGACAGCCTTGCAGGTC-3’	65
*Ifnb*	For: 5’-AACCTCACCTACAGGGCGGACTTCA-3’; Rev: 5’-TCCCACGTCAATCTTTCCTCTTGCTTT-3’	65

### DNA isolation and quantification of AAV genome copies

Liver fragments were collected, snap-frozen in liquid nitrogen and stored at -80°C. For quantification of AAV genomes, DNA was isolated using phenol-chloroform-isoamyl alcohol extraction method. First, liver fragments were homogenized using Tissue Lyzer in lysis buffer (10 mM EDTA, 1% SDS and 400 μg/mL Proteinase K in volume of 500 μl adjusted with tris buffered saline) and incubated for 1 hour at 37°C to disintegrate the tissue. Obtained solution was later combined with equal volume of phenol-chloroform-isoamyl alcohol (25:24:1, Sigma-Aldrich) and mixed vigorously for 1 minute and then centrifuged for 10 minutes at 12 000×*g*. Top, aqueous phase was collected to fresh tubes and 10 μl of 5 M NaCl and 1 ml of absolute ethanol were added. Then, samples were left at -20°C overnight for precipitation. The following day, after 15 minutes of centrifugation at 12 000×*g*, DNA pellet was washed once with 70% ethanol, dried and resuspended in ultrapure water. AAV genome copies were quantified with qPCR using ITR-binding TaqMan probe, as in case of titration procedure.

### Immunofluorescence

For immunofluorescent detection of macrophages liver sections were stained against F4/80 and CD11b antigens. Paraffin-embedded liver sections were deparaffinized and subjected to antigen retrieval using 0.05 M sodium citrate buffer (pH 6.0) in microwave oven. Tissues were washed in PBS and blocked in a solution containing 10% goat serum. After blocking, tissue sections were incubated with primary rat antibodies recognizing mouse F4/80 antigen (dilution 1:200, overnight at 4°C; clone: BM8; eBioscience) followed by fluorochrome-conjugated secondary anti-rat antibody (Alexa Fluor 488; dilution 1:400, 1 hour 30 minutes at RT). Liver cryosections were incubated with rat anti-mouse CD11b antibody (dilution 1:200, overnight, 4°C; clone: 5C6; Bio-Rad) followed by fluorochrome-conjugated secondary anti-rat antibody (Alexa Fluor 488; dilution 1:400, 1 hour 30 minutes at RT). All sections were mounted with fluorescence mounting medium (Dako) and analyzed with a fluorescent microscope (Nikon) at 400× magnification.

### Analysis of inflammatory cytokines with FlexMap3D Luminex system

Liver fragments were homogenized in lysis buffer (50mM Tris-HCl pH 8.0, 150 mM NaCl, 1% NP-40, 0.5% Na-deoxycholate, 0.1% SDS) with protease inhibitors (cOmplete™ EDTA-free Protease Inhibitor Cocktail, Roche) using Tissue Lyzer (Qiagen). The samples were incubated on ice for 5 minutes and cleared by centrifugation (12 000×*g*, 10 minutes, 4°C). The supernatant was collected and stored in -80°C. Analysis was performed using Mouse Cytokine Magnetic 10-Plex Panel (Invitrogen) according to the manufacturer’s instructions. The results were analyzed with FLEXMAP 3D xPONENT software.

### ELISA

The content of mouse IL-6 in plasma was evaluated according to vendor’s protocol.

### Determination of AAV neutralizing antibodies (transduction-based assay)

Plasma samples collected from mice were first incubated for 30 minutes at 56°C to inactivate components of the complement system that may interfere with the assay. Then, serial dilutions (2, 4, 8, 16 for one dose of AAV vectors; 20, 40, 80, 160 and 800 for two doses of AAVs) of plasma in FBS were prepared in a final volume of 20 μl. Next, 1 μl of 1×10^9^ AAV particles was added to each sample and such mixture was incubated for 3 hours at 37°C to allow binding of the antibodies to AAV capsids. In case of a positive control, vectors were preincubated with FBS only. Subsequently, neutralized vectors were used for transduction of 1×10^5^ HEK293 at the time of seeding the cells on 24-well plates. Transgene expression was analyzed after 96 hours using flow cytometer. Briefly, cells were washed once with PBS and detached from the culture surface with 0.05% trypsin solution (Gibco). Next, 1 ml of DMEM HG supplemented with 10% FBS was added and cells were centrifuged for 5 minutes at 350×*g*. Pellet was resuspended in PBS with 2 mM EDTA and 0.2 μg/ml DAPI to exclude nonviable cells from further analysis with LSRFortessa cytometer.

### Isolation and purification of KCs

KCs were isolated and purified according to the method described previously [37]. Mice were subjected to pharmacological euthanasia through intraperitoneal administration of a mixture of ketamine hydrochloride (200 mg/kg) and xylazine hydrochloride (40 mg/kg). Then, liver was perfused *in situ* through left ventricle punctured with PE-50 catheter connected to a perfusion apparatus (3 ml/minute flow rate) with 10 ml of 0.5 U/ml heparin in PBS without calcium and magnesium ions and subsequently with 10 ml of 0.1% collagenase type IV in HBSS buffer. Afterwards, the liver was excised, transferred to collagenase solution and after disruption of Glisson’s capsule it was cut into smaller pieces to allow more efficient tissue digestion. Samples were incubated for 10 min at 37°C and then for 1 hour at 25°C. Obtained suspension was passed through 100 μm nylon strainer and centrifuged for 5 minutes at 300×*g*. Cells were washed once with PBS and resuspended in RPMI medium. Next, differential centrifugation (3 minutes, 50×*g*, 4°C) was performed in order to separate parenchymal cells from KCs. After centrifugation KCs were present in the supernatant, while parenchymal fraction was in the pellet. KCs were further purified though selective adhesion, where unattached cells were removed from the culture 2 hours after seeding. Primary culture of KCs was maintained in DMEM HG supplemented with 10% FBS and antibiotics.

### Stimulation of KCs with TLR9 ligand

KCs were stimulated with 5 μM class A CpG oligonucleotide (ODN 1585, InvivoGen) in DMEM HG supplemented with 10% FBS and antibiotics. After 24 hours cells were collected for RNA isolation and gene expression analysis.

### Confocal microscopy for detection of AAV9 in KCs

KCs were seeded on glass culture surface and 24 hours later exposed to scAAV9-empty vectors at a multiplicity of infection (MOI) of 10^4^ viral genomes (vg)/cell. Next, after 1 hour of incubation at 37°C and subsequent incubation for 1 hour at 4°C, cells were fixed with 4% paraformaldehyde, washed with PBS and permeabilized with 0.1% Triton X-100. In the next step, to reduce non-specific antibody binding, blocking buffer (5% BSA, 0.5% Tween 20, 10% goat serum in PBS) was added for 1 hour at room temperature and subsequently, it was replaced with mouse anti-AAV9 antibody (ADK9, Progen) diluted 10 times in PBS containing 0.5% BSA, 0.05% Tween 20 and 1% goat serum for 2 hours at RT. Then, cells were washed with PBS and incubated with fluorochrome-conjugated goat anti-mouse IgG, IgM, IgA secondary antibody (AlexaFluor 488; dilution 1:200, 1 hour at RT). Slides were sealed with mounting medium containing DAPI and analyzed with Leica TCS SP5 II microscope together with LAS AF software (Leica Microsystems).

### Statistical analysis

Results are presented as mean value ± SEM, unless otherwise stated. Statistical analyses were carried out using GraphPad Prism software version 6 (GraphPad Software, Inc.). For two-group comparisons, Mann-Whitney U test was applied. For comparing more than two groups, a one-way ANOVA followed by Tukey’s post hoc test was applied. For * p>0.05, for ** p>0.01 and for *** p>0.001.

## Results

### Hepatic expression of Mcp-1 is significantly affected by the lack of HO-1, but not much changed upon scAAV9 vectors delivery

**Fig 1:**
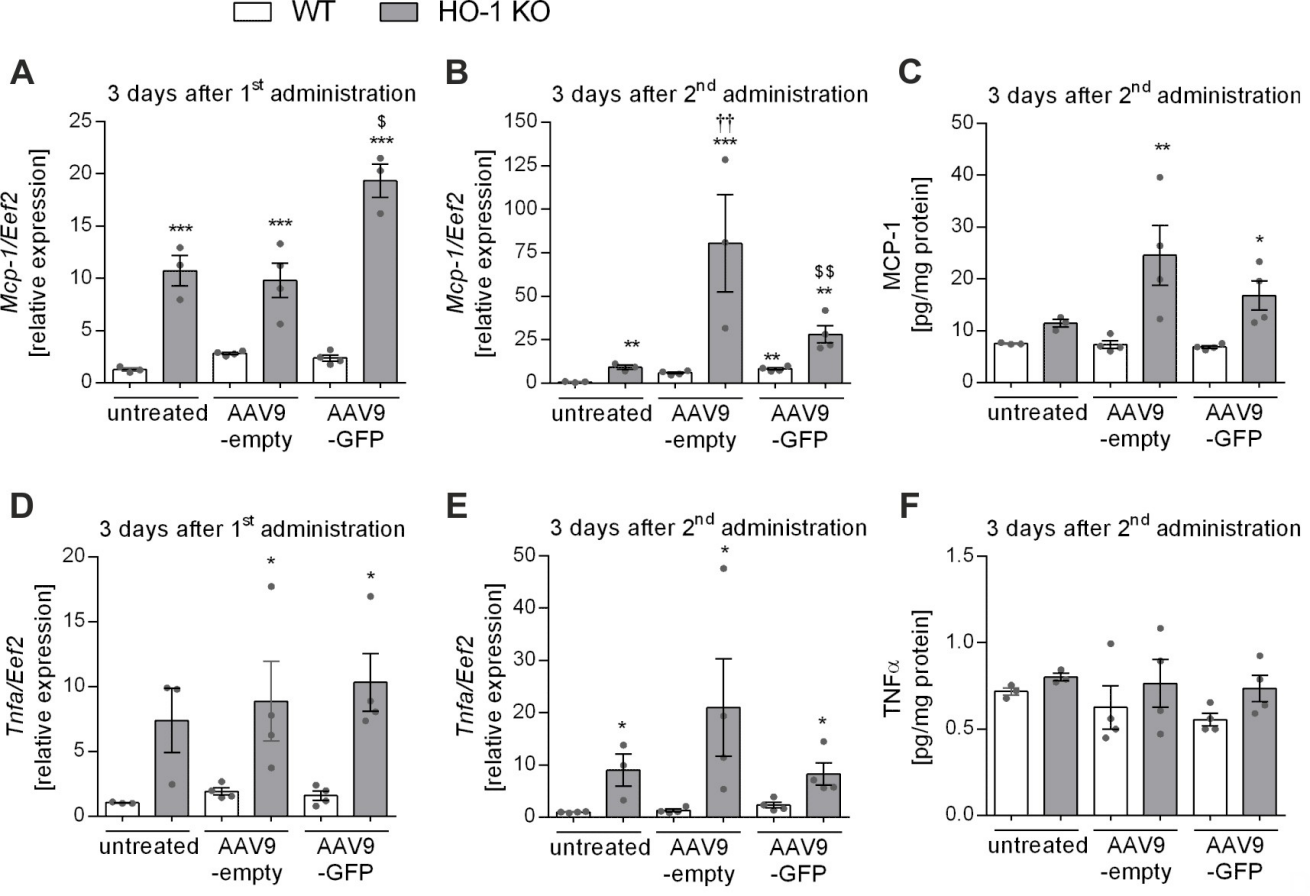
Hepatic expression of inflammatory genes in WT and HO-1 KO mice after scAAV9 vectors injection. (A) The qPCR for *Mcp-1* 3 days post-1^st^ injection of scAAV9 vectors. (B) The qPCR for *Mcp-1* 3 days after scAAV9 re-administration. (C) The Luminex assay for MCP-1 protein 3 days after scAAV9 re-administration. (D) The qPCR for *Tnfa* 3 days post-1^st^ injection of scAAV9 vectors. (E) The qPCR for *Tnfa* 3 days after scAAV9 re-administration. (F) The Luminex assay for TNFα protein 3 days after scAAV9 re-administration. Values represent means ± SEM (n = 3-4/group). p<0.05 * vs. appropriate WT, † vs. appropriate untreated, $ vs. appropriate scAAV-empty.

It was previously shown that scAAV vectors are capable of inducing TLR9-mediated increase in the expression of IFNα/β, MCP-1, TNF-α, and other proinflammatory cytokines and chemokines in the liver [17]. However, such innate immune response to scAAV was self-limited and resolved within less than 12 hours after vector administration [17]. In relation to the onset of transgene expression from scAAV vectors [2] and temporal kinetics of monocyte/macrophage infiltration in response to a potential damaging stimuli [38], our analyses were performed 3 days after the 1^st^ and 2^nd^ vector dose. We observed slight (about 2 times) upregulation of *Mcp-1* expression in the livers of WT mice 3 days post-1^st^ injection of both (empty and GFP-expressing) scAAV9 vectors (Fig 1A). Regardless of the treatment, *Mcp-1* expression in the livers of HO-1 KO mice was markedly higher than in WT mice (Fig 1A). Moreover, higher expression of *Mcp-1* was detected in HO-1 KO mice injected with one dose of scAAV9-GFP when compared to untreated control or scAAV9-empty vector (Fig 1A). The induction of *Mcp-1* in WT mice 3 days after the 2^nd^ dose of scAAV9-empty or scAAV9-GFP was even more pronounced than after the 1^st^ vector dose (5-fold and 7-fold change, respectively) when compared to untreated control (Fig 1B). In HO-1 KO mice we also observed induction of *Mcp-1* expression 3 days after re-administration of either scAAV9-empty or scAAV9-GFP (6-fold and 3-fold change, respectively) when compared to untreated controls (Fig 1B). MCP-1 protein level was higher in the livers of HO-1 KO than of WT mice (Fig 1C) and there was a slight, but statistically not significant increase in MCP-1 protein level in HO-1 KO mice 3 days after re-administration of either scAAV9-empty or scAAV9-GFP as compared to untreated HO-1 KO control (Fig 1C).

The expression of *Tnfα* was considerably higher in the liver of HO-1 KO than of WT mice, but in both genotypes there were no differences after one or two doses of scAAV9 vectors when compared to untreated control (Fig 1D and 1E). In both genotypes the TNFα protein levels also remained unchanged after scAAV9 vector delivery (Fig 1F). Hepatic expression of other analyzed cytokines (IL-1β, IL-10, IL-6, IL-12, IL-17, IFNα/β, IFNγ, MIP-1α) did not differ between both genotypes and treatments (not shown).

### Phenotypic analysis of hepatic macrophages reveals differences between WT and HO-1 KO mice

**Fig 2:**
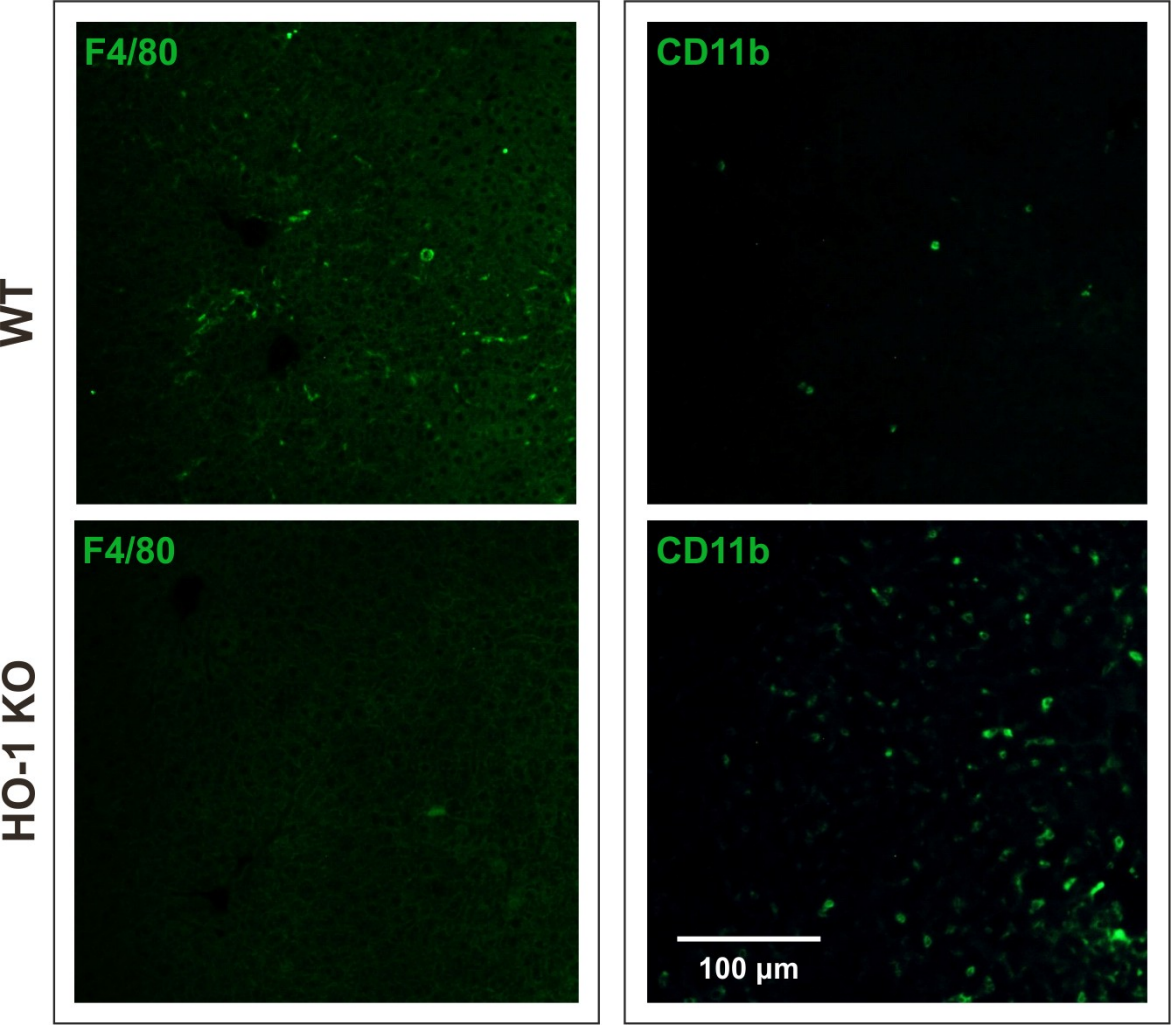
Immunofluorescent staining of macrophages in the livers of WT and HO-1 KO mice. Representative fluorescent images of F4/80 marker (green, left panel). Representative fluorescent images of CD11b marker (green, right panel). Scale bar = 100 μm.

**Fig 3:**
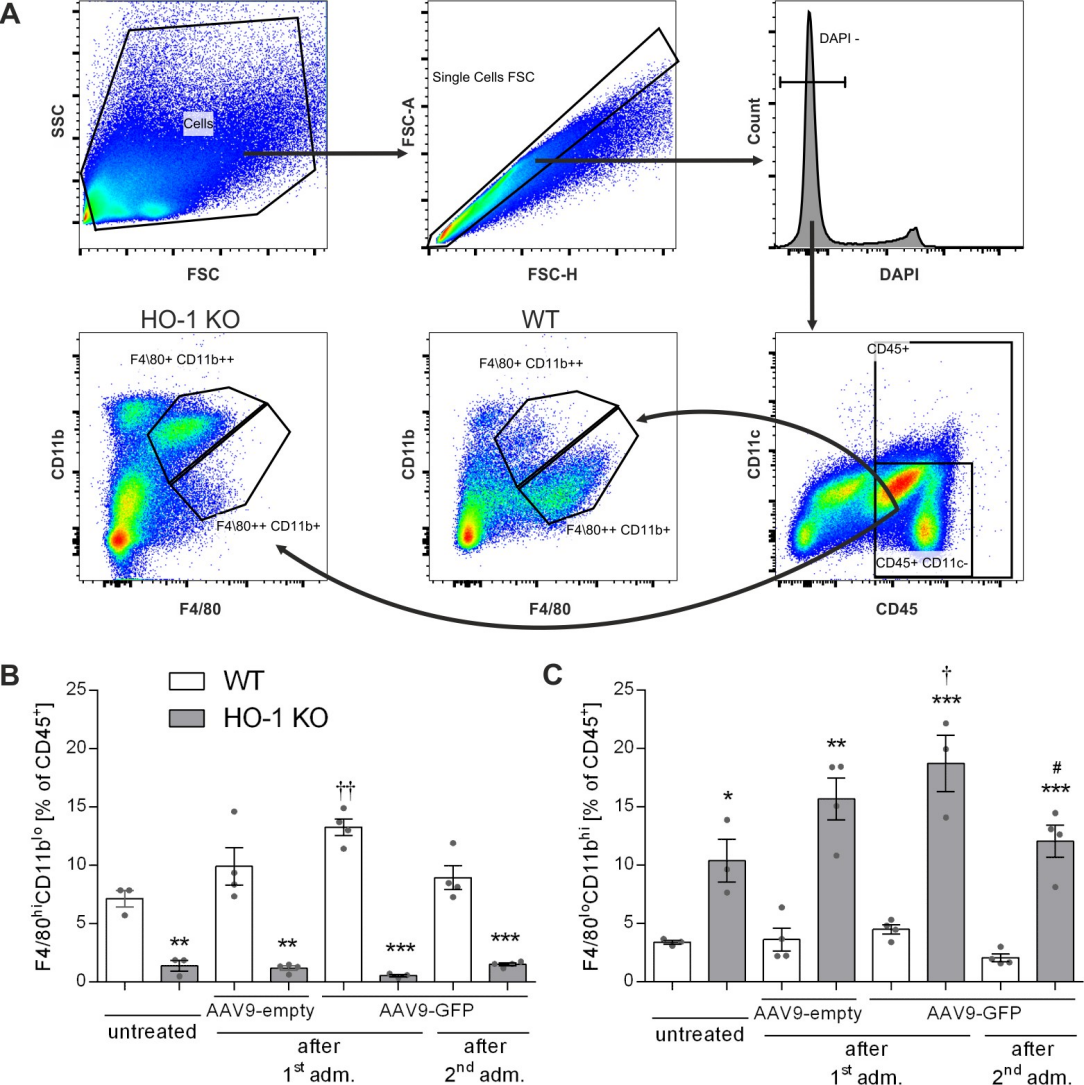
Flow cytometric analysis of two populations of hepatic macrophages in WT and HO-1 KO mice after scAAV9 vectors injection. (A) Gating strategy and representative dot plots demonstrating F4/80^low^CD11b^high^ (MDMs) and F4/80^high^CD11b^low^ (KCs). Flow cytometric analysis of KCs (B) and MDMs (C) 3 days after first and 3 days after second administration of scAAV9 vectors. Values represent means ± SEM (n = 3–4 mice/group). *p < 0.05 vs. appropriate WT; † p < 0.05 vs. appropriate untreated; # p < 0.05 vs. AAV9-GFP after the 1^st^ administration.

In healthy adult mice KCs represent about 35% of the non-parenchymal liver cells [39]. Their specific localization and the fact that they constitute 80–90% of tissue macrophages present in the body suggest a central role of the liver in systemic and local defence [39]. It was previously shown that in the absence of HO-1 KCs die following phagocytosis of senescent red blood cells due to toxicity of the accumulated heme [29]. Our immunohistochemical analysis revealed that the frequency of F4/80 expressing cells in the liver of HO-1 KO mice is lower than in WT mice (Fig 2; left panel). On the other hand, the liver of HO-1 KO mice contained higher number of CD11b-positive cells when compared to WT mice (Fig 2; right panel), what is in line with previous observation [30]. KCs identification is based mainly on the expression of F4/80 marker, sometimes combined with CD11b and/or CD68 [40]. More recently, flow cytometric analysis by Movita *et al*. defined two populations of hepatic macrophages: F4/80^low^ CD11b^high^ (MDMs) and F4/80^high^ CD11b^low^ (KCs) [41]. We applied similar strategy to quantitatively analyze the numbers of these cells in the livers of WT and HO-1 KO mice (Fig 3A). We found that the frequency of KCs in adult untreated WT mice is about 7% of the CD45^+^ leukocytes (Fig 3B). Independently of the treatment, the number of KCs were several times higher in WT than in HO-1 KO mice and only in case of WT mice their number significantly increased 3 days after delivery of the 1^st^ dose, but not after the 2^nd^ injection of scAAV9-GFP (Fig 3B). On the contrary, the livers of adult HO-1 KO mice contained several times more MDMs than their WT littermates (Fig 3C), and only in HO-1 KO mice this population expanded 3 days after the 1^st^, but not after the 2^nd^ dose of scAAV9-GFP (Fig 3C). These data indicate a lack of considerable macrophage infiltration into the liver upon scAAV9 re-administration independently of the initial liver KC/MDM numbers.

### Lack of HO-1 results in higher AAV9 neutralizing activity and lower transgene expression in two populations of liver macrophages

**Fig 4:**
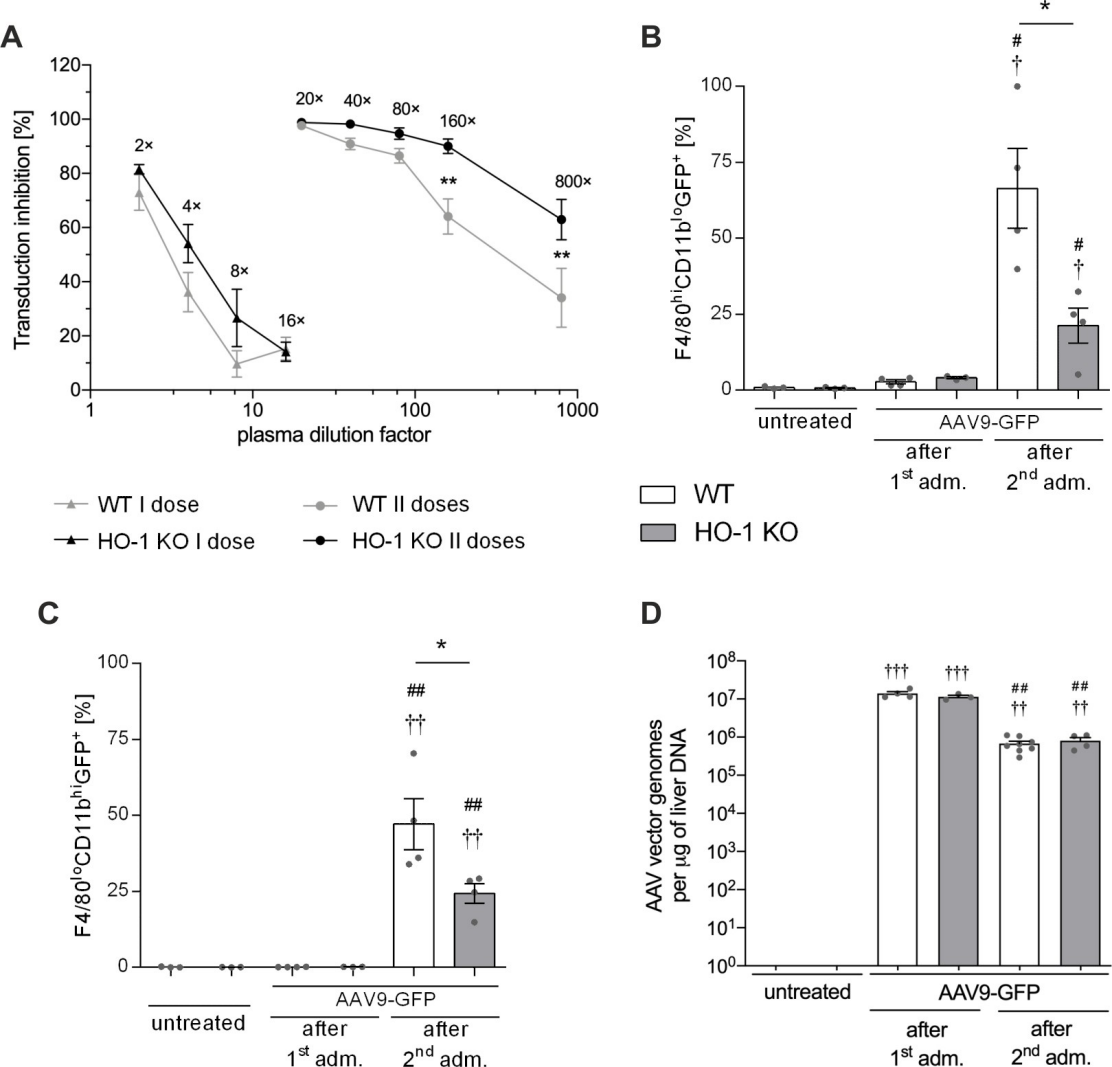
Plasma neutralizing activity and transgene expression in liver macrophages after scAAV9-GFP transduction. (A) Virus neutralization test in plasma samples collected from WT and HO-1 KO mice 3 days after the 1^st^ administration and 3 days after the 2^nd^ administration of scAAV9-GFP. Flow cytometric analysis of GFP-positive (GFP^+^) KCs (B) and MDMs (C) in the livers of WT and HO-1 KO mice 3 days after the 1^st^ administration and 3 days after the 2^nd^ administration of scAAV9-GFP. (D) The qPCR assessing the number of vector genomes in the livers of WT and HO-1 KO mice 3 days after the 1^st^ administration and 3 days after the 2^nd^ administration of scAAV9-GFP. Values represent means ± SEM (n = 3-4/group). *p < 0.05 vs. appropriate WT; † p < 0.05 vs. appropriate untreated; # p < 0.05 vs. AAV9-GFP after the 1^st^ administration.

The *in vitro* neutralization assay revealed that inhibition of HEK293 transduction with plasma samples collected 3 days post-1^st^ injection of scAAV9-GFP was very limited, without significant differences between WT and HO-1 KO mice (Fig 4A). In another experiment using different control scAAV9 vector (without GFP expression), we observed several times higher and comparable between both genotypes neutralizing activity of the plasma collected 14 days after the 1^st^ vector dose (not shown). After re-administration of scAAV9-GFP we still detected very effective inhibition of HEK293 cells transduction confirming efficient generation of nAbs to AAV9 vectors (Fig 4A). Interestingly, this time stronger inhibitory effect was observed in HO-1 KO individuals–comparable transduction efficiency was achieved for 160-times diluted plasma of WT and 800-times diluted plasma of HO-1 KO mice, indicating about 5 times higher neutralizing activity of HO-1 KO mice-derived plasma (Fig 4A). Humoral immunity to AAV vectors was recently associated with increased secretion of IL-1β and IL-6 cytokines by monocyte-related dendritic cells [42]. Here, we found considerably higher level of IL-6 in the plasma of HO-1 KO when compared to WT mice following exposure to the 2^nd^ dose of AAV9 (S1 Fig).

Three days after vector re-administration, the livers of WT mice contained significantly more GFP-positive KCs (Fig 4B) and MDMs (Fig 4C) than the livers of HO-1 KO mice. Similarly, 3 days after vector re-administration the percentage of GFP-positive cells within a heterogenous population of CD45-negative liver cells was about 2 times higher in WT than in HO-1 KO mice (not shown). Different numbers of GFP-expressing macrophages in WT and HO-1 KO mice could not be explained by differences in availability of AAV9 primary receptor (the terminal galactose in N-linked glycans) [43]. The staining of both populations of hepatic macrophages with *Maackia Amurensis* lectin-II (MAL-II; binds sialic acid attached to terminal galactose in α-2,3 linkage) or with *Sambucus Nigra* agglutinin (SNA; binds sialic acid attached to terminal galactose in α-2,6 linkage) resulted in comparable fluorescence intensity (S2 and S3 Figs, respectively). Moreover, after the 1^st^ as well as after the 2^nd^ vector dose we detected comparable numbers of AAV genomes in the livers of WT and HO-1 KO mice (Fig 4D) indicating similar transduction efficiency between genotypes.

### AAV9 infects the primary culture of Kupffer cells without triggering TLR9-mediated immune responses, but does not lead to transgene expression

**Fig 5:**
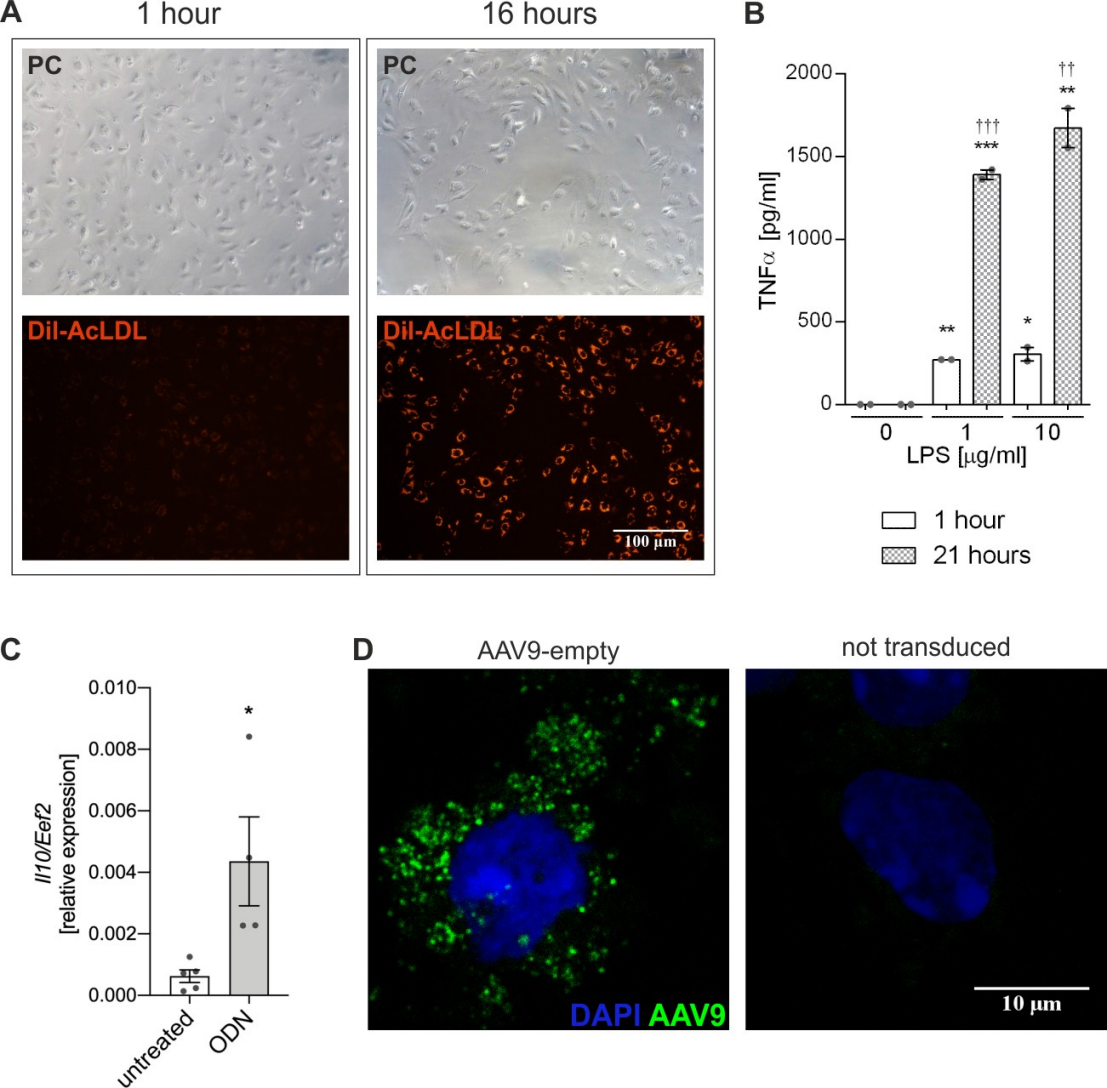
Functional assessment of mouse KCs in culture. (A) Phase contrast and fluorescence microscopy images of KCs fluorescently labelled with DiI-AcLDL after 1 hour and 16 hours of incubation. (B) Production of TNFα in the supernatant of KCs stimulated with LPS for 1 hour and 21 hours in FBS-free conditions. (C) The qPCR for *Il10* after 24 hours stimulation with CpG ODN 1585. (D) Confocal microscopy images demonstrating internalization of scAAV9 particles by KCs 2 hours after addition of 10^4^ MOI of scAAV9-empty (left panel). * p < 0.05 vs. appropriate untreated control; † p < 0.05 vs. appropriate 1 hour treatment.

Due to specific localization and function in the liver, Kupffer cells are indispensable for organ's response to foreign agents, such as bacteria or viruses [39]. Thus, in order to more deeply investigate their potential responses to scAAV9 vectors, we isolated and purified the cells according to the previously described method [37]. First, the cells were subjected to two functional tests: uptake of DiI-labelled acetylated low-density lipoproteins (DiI-AcLDL) and production of inflammatory cytokine TNFα in response to LPS. The internalization of fluorescently-labelled AcLDL, which in macrophages is driven by a family of scavenger receptors (SRs), including SR-A, SR-B1, and CD36 [44], was visible as early as 1 hour after addition to the cell culture media (Fig 5A, left panel) and after 16 hours nearly all cells exhibited strong fluorescence (Fig 5A, right panel). Next, KCs were stimulated for 1 or 21 hours with different concentrations of bacterial endotoxin (LPS). At both time-points we have observed LPS-induced production of TNFα (Fig 5B). These results demonstrated that, in line with previous observations [37], the isolated cells had ability to uptake the modified AcLDL and to produce specific inflammatory cytokine in response to LPS. Additionally, we detected increased expression of *Il10* in cells stimulated with CpG ODN (Fig 5C, S4 Fig) and thus, confirmed the previous observations [41] that KCs *in vitro* have functional signalling in response to TLR9 agonist.

**Fig 6:**
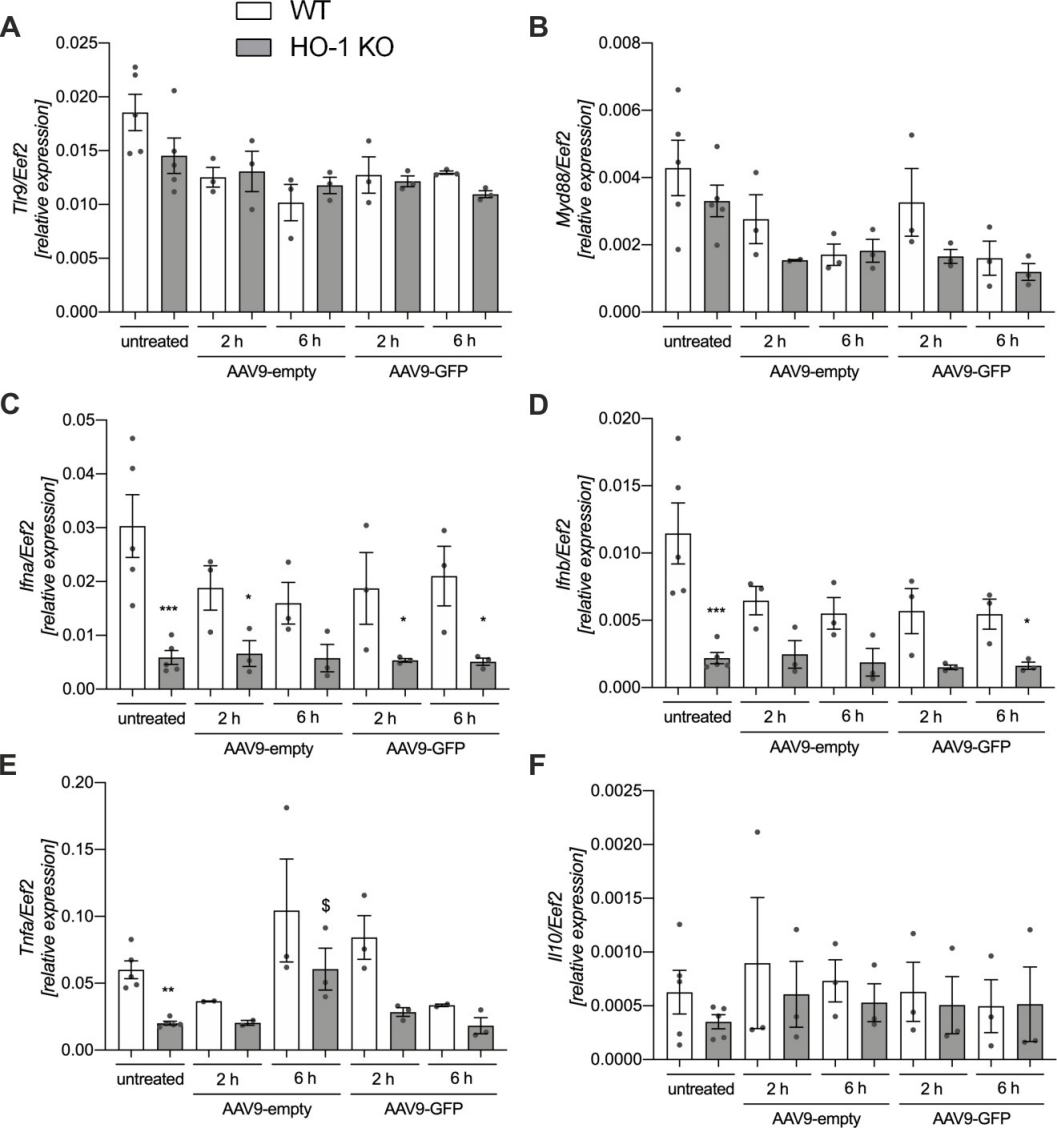
Expression of genes associated with TLR9 signaling in primary culture of KCs exposed to scAAV9 vectors. The qPCR for (A) *Tlr9*, (B) *Myd88*, (C) *Ifna*, (D) *Ifnb*, (E) *Tnfa* and (F) *Il10* at 2 and 6 hours after *in vitro* exposure of WT and HO-1 KO KCs to 10^4^ MOI of scAAV9-empty or scAAV9-GFP. * p < 0.05 vs. appropriate WT; $ p < 0.05 vs. untreated HO-1 KO.

Next, we exposed these cells to scAAV9-empty (MOI 10^4^). Already after 2 hours following addition of scAAV9-empty to the cultured KCs, the vector particles were present in the cytoplasm (Fig 5D, left panel). AAV vectors were previously shown to activate inflammatory genes in liver within 2 hours after gene transfer *via* NF-κB pathway and TLR2- or TLR9-mediated recognition of capsid or vector DNA, but these innate immune responses were attenuated in the next 12 hours [17–19]. In our cultured WT and HO-1 KO KCs the expression of genes associated with TLR9 signalling—*Tlr9*, *Myd88*, *Ifnα*, *Ifnβ*, *Tnfα* and *Il10 –*remained unchanged 2 and 6 hours following addition of scAAV9-empty or scAAV9-GFP (Fig 6A, 6B, 6C, 6D, 6E and 6F, respectively). Moreover, unlike the *in vivo* infection, GFP transgene was not expressed in the primary culture of KCs exposed to scAAV9-GFP, as monitored up to 7 days after addition of scAAV9-GFP (MOI 10^4^, not shown).

## Discussion

Data presented here show that the exposure of mice to two doses (separated by 3 months) of scAAV9 did not lead to a considerable increase in inflammatory cytokines expression and macrophage infiltration, neither in WT nor in HO-1 KO mice. However, HO-1 deficiency was associated with notably decreased transgene expression in two main populations of hepatic macrophages–MDMs and KCs. In addition, KCs in culture internalized scAAV9 vectors, but did not express the transgene and did not exhibit any signs of TLR9-mediated immune response activation.

Application of scAAV instead of ssAAV was previously shown to cause a substantial increase in the innate TLR9-dependent immune responses in liver independently of the strain of mouse or the transgene/promoter cassette [17]. TLR9 is located in endosomes and recognizes unmethylated cytosine-guanine dinucleotide motifs that are typically present in viral or bacterial, but not mammalian DNA [45]. The downstream signalling, through the MyD88 adapter molecule, activates NF-κB and consequently the expression of proinflammatory cytokines and induces the expression of type I interferons (IFNα and β) via IFN-regulatory factors (IRF-3 and -7). Martino *et al*. demonstrated the TLR9-mediated induction of IFNα/β, MCP-1, TNF-α, and other proinflammatory cytokines and chemokines in the liver by scAAV vectors [17]. Importantly, the innate response to scAAV2 was self-limited and resolved within less than 12 hours after vector administration [17]. Cells of the monocytic lineage are the primary synthesizers of TNFα, a powerful pro-inflammatory agent that regulates many facets of macrophage function [46]. In our study, the mRNA expression of this cytokine, similarly to *Mcp-1*, was higher in HO-1 KO than in WT mice. Interestingly, unlike MCP-1, TNFα protein level did not correspond with its mRNA. The high potency of TNFα in terms of protecting the host from infection requires stringent control of this cytokine expression. An important regulatory checkpoint is AU-rich elements (AREs) embedded in the 3′ UTR of TNFα mRNA offering protection against the overexpression [47]. Perhaps, a tight regulation of TNFα mRNA translation might explain the observed here by us discrepancy between this cytokine mRNA and protein level in HO-1 KO mice. In addition, unlike *Mcp-1*, the expression of *Tnfα* was increased neither 3 days after the 1^st^ scAAV9 dose nor 3 days upon vector re-administration.

It was previously shown that in the liver AAV-mediated induction of TNFα, as well as IL-1β and IL-6 expression, precedes MCP-1 [48] indicating a possible mechanism of activation of MCP-1 transcription by these cytokines [49]. MCP-1 is produced by many cell types, but monocytes/macrophages are its major source [30, 39]. Moreover, MCP-1 is known to attract memory T cells expressing CCR2 and influence T-cell immunity [28,35]. In the study by Martino *et al*. the production of MCP-1 in response to scAAV vectors was to some extent dependent on KCs as inactivation of these cells with GdCl_3_ administration partially blocked macrophage infiltration in the liver [17]. In our HO-1 KO mice, characterized by decreased numbers of KCs, the expression of *Mcp-1* was increased 3 days after the 2^nd^ dose of scAAV9, but the protein levels and abundance of macrophages were not affected. This, together with the lack of activation of TLR9 downstream target genes, indicates the minor potential of scAAV vectors to activate innate immune responses in the liver regardless of the initial KCs/MDMs ratio. Still, we cannot rule out transient upregulation of proinflammatory transcripts in the liver at earlier time points, within first hours after scAAV administration as described by Martino et al [17]. Nevertheless, it would require further investigation since the studies published so far focused on AAV2 serotype, which has much shorter clearance half-life than AAV9 after intravenous administration [50]. More rapid uptake of the vector by the cells may not only induce a stronger immune response but also contribute to different kinetics of the whole process depending on the investigated serotype.

Under inflammatory conditions circulating bone marrow-derived monocytes contribute to the repopulation of the emptied niches of hepatic KCs [12, 13]. Fate-mapping experiments revealed efficient refilling starting from day 4 post-infection and a gradual differentiation of bone marrow-derived monocytes into F4/80^high^CD11b^low^ cells [13]. HO-1 deficient mice exhibit chronic inflammation demonstrated by high peripheral white blood cell counts, increased monocyte vascular adhesion and tissue infiltration [51, 52]. We have recently shown higher steady-state numbers of pro-inflammatory Ly6C^hi^ monocytes in the blood of mice lacking HO-1 [53]. Moreover, after myocardial infarction the increased expression of *Mcp-1* in these animals was associated with increased infiltration of Ly6C^hi^ monocytes into the cardiac muscle, what resulted in adverse remodeling and significantly impaired cardiac function [53]. In the injured liver the accumulation of inflammatory Ly6C^hi^ monocytes also depends on the interaction of MCP-1 with its receptor CCR2 [54]. Increased level of MCP-1 in the livers of HO-1 KO mice resulted in constant recruitment and significantly higher than in WT mice numbers of F4/80^low^CD11b^high^ MDMs. It was recently shown that WT bone marrow derived macrophages engraft in the livers of HO-1 KO mice, differentiate into KCs, and self-renew, protecting the recipient for several weeks and potentially for a lifetime [29, 55]. In the present study, in WT mice we noted the expansion of KCs 3 days after delivery of the 1^st^ dose of scAAV9-GFP (their number after the 2^nd^ vector dose was comparable with untreated mice). On the other hand, in the livers of HO-1 KO mice we noted the expansion of MDMs 3 days after the 1^st^ dose of scAAV9-GFP (their number after the 2^nd^ vector dose was comparable with untreated mice). Previous study demonstrated that circulating monocytes engraft in the liver and become long-lived self-renewing Kupffer cells [12]. Thus, it is possible that in response to the 1^st^ scAAV9-GFP transduction monocytes have been recruited to the liver of both WT and HO-1 KO mice, but for example due to the niche specificity or lack of HO-1, only MDMs in WT mice were able to differentiate towards KCs resulting in these cells expansion. Another reason for KCs expansion in WT mice could be their local proliferation in response to scAAV9-GFP transduction. Both hypotheses would require further investigation using specific lineage tracing approach.

In the present study, we observed significantly higher AAV9 neutralizing activity of the plasma of HO-1 KO than of WT mice 3 days after scAAV9-GFP re-administration. Neutralizing antibody formation can be influenced by the genetic background of the host [56]. Several lines of evidence suggest that HO-1 exerts immunomodulatory effects not only on innate immune responses, but also on adaptive immunity [28]. In the field of transplantation, HO-1 was shown to regulate B cell infiltration into tissues and control destructive allo-antibody responses [52, 57]. In addition, hemophilia A patients with a higher frequency of alleles with large (GT)_n_ repeats (n≥30) within *HMOX1* promoter (associated with lesser HO-1 expression [33, 58]), had an increased prevalence of development of inhibitory anti-factor VIII antibodies [59]. A recent study investigating antibody formation against AAV in healthy humans found that the AAV capsid activates IL-1β and IL-6 cytokine secretion in monocyte-related dendritic cells and that IL-1β and IL-6 blockade inhibited the anti-capsid humoral response *in vitro* and *in vivo* in mice [42]. The study also demonstrated that in subjects previously exposed to WT AAV both IL-1β and IL-6 cytokines triggered the differentiation of capsid-specific memory B cells into antibody-secreting cells and anti-capsid antibody production [42]. In our study, the plasma level of IL-6 was considerably higher in HO-1 KO than in WT mice re-exposed to scAAV9-GFP. Moreover, 14 days after 1^st^ administration of scAAV9 there was no difference in AAV9 neutralizing activity between both genotypes (not shown). This indicates that upon re-exposure to antigen HO-1 expression may influence the AAV-nAbs production, but the exact mechanism considering B- and T-cells function requires further exploration.

Due to their specific location, KCs are directly exposed to any pathogens entering the liver with blood and were shown to be involved in both immunogenic and tolerogenic immune reactions [14–16]. As soon as 3 days after the 1^st^ scAAV9-GFP vector dose we observed a small number of KCs (but not MDMs) expressing GFP in both WT and HO-1 KO mice. This indicates that KCs were the primary cells in the liver exposed to AAVs. Apparently, 3 days after scAAV9-GFP vector re-administration, the numbers of GFP-expressing cells within two populations of hepatic macrophages were much higher in WT than in HO-1 KO mice. The staining of both populations of hepatic macrophages with MAL II and SNA demonstrated that the differences in GFP expression between WT and HO-1 KO mice could not be explained by different availability of AAV9 primary receptor (the terminal galactose in N-linked glycans) [43]. High titers of nAbs can directly contribute to the lower transgene expression. However, higher AAV9 neutralization activity of plasma from HO-1 KO when compared to WT mice was observed after the 2^nd^ but not after the 1^st^ vector dose. Moreover, a similar number of vector genomes detected in the livers of WT and HO-1 KO mice indicates comparable transduction efficiency in both genotypes. Thus, other factors could contribute to the decreased number of GFP-expressing cells in HO-1 KO mice such as faster cell turnover rate or capsid-specific cytotoxic T lymphocyte responses [21].

In contrary, our *in vitro* data demonstrate lack of transgene expression in cultured KCs exposed to AAV9-GFP. We confirmed that although vectors are taken up by the cells, transgene is not expressed. A recent paper by Fitzpatrick *et al*. demonstrated the presence of AAV binding antibodies (bAbs) in adult human subjects [60]. Since the non-neutralizing AAV-bAbs can positively impact gene transfer, we wanted to assess whether they might facilitate the *in vitro* transduction of KCs (e.g. *via* influencing the uptake or trafficking of AAV vectors inside the cells). For this, we pre-incubated the scAAV9-GFP vectors with mouse plasma containing AAV9 antibodies, however, we still did not detect transgene expression in cultured KCs (not shown). Thus, it seems that some other factors, apparently absent *in vitro*, may be necessary for transgene expression in KCs. Upon intravenous delivery AAVs may interact with other, apart from neutralizing or binding antibodies, blood components (i.e. galectin 3 binding protein, C-reactive protein or platelet factor-4), which can also alter the vectors’ therapeutic efficiencies [61]. Thus, analysis of the composition of the mouse sera would be necessary to verify the impact of different serum proteins on KCs transduction by AAV9. The expression of genes associated with TLR9 signalling did not differ in response to scAAV. This is in agreement with other data indicating that AAVs in the liver activate rather plasmacytoid dendritic cells (pDCs) than non-pDCs such as KCs, to produce type I interferons through the TLR9-MyD88 pathway [18].

In summary, our data demonstrate low potential of scAAV9 to stimulate inflammatory response at the analysed time points regardless of the presence or absence of HO-1 and initial numbers of KCs/MDMs in the liver. On the other hand, 3 days after vector re-administration we observed higher AAV9 neutralizing activity in plasma and significantly lower transgene expression in hepatic macrophages of HO-1 KO mice. This suggests that different level of HO-1 expression, in humans determined by the polymorphisms in the *HMOX1* promoter, may confer different genetic predispositions to the induction of the immune responses against AAV vectors and in this way affect their therapeutic potential.

## Supporting information

S1 Fig Interleukin 6 in plasma of WT and HO-1 KO mice after AAV9 administration. ELISA assessment of IL-6 in plasma collected from untreated mice, 3 days after 1^st^ administration and 3 days after 2^nd^ administration of scAAV9-GFP.
(TIF)

S2 Fig Flow cytometric analysis of *Maackia amurensis* lectin-II binding in two populations of hepatic macrophages in WT and HO-1 KO mice. *Maackia amurensis* lectin-II **(**MAL-II) binds sialic acid attached to terminal galactose in α-2,3 linkage. (A) Percentage of F4/80^high^CD11b^low^ cells labelled with MALII and (B) their median fluorescence intensity. (C) Percentage of F4/80^low^CD11b^high^ cells labelled with MALII and (D) their median fluorescence intensity.
(TIF)

S3 Fig Flow cytometric analysis of *Sambucus nigra* agglutinin binding in two populations of hepatic macrophages in WT and HO-1 KO mice. *Sambucus nigra* agglutinin (SNA) binds sialic acid attached to terminal galactose in α-2,6 linkage. (A) Percentage of F4/80^high^CD11b^low^ cells labelled with SNA and (B) their median fluorescence intensity. (C) Percentage of F4/80^low^CD11b^high^ cells labelled with SNA and (D) their median fluorescence intensity.
(TIF)

S4 Fig Expression of genes associated with TLR9 signaling in primary culture of KCs following stimulation with ODN. The qPCR for (A) *Tlr9*, (B) *Myd88*, (C) *Tnfa*, (D) *Ifna*, (E) *Ifnb*, and (F) *Il10* at 24 h after *in vitro* stimulation of WT and HO-1 KO KCs with ODN 1585. *p < 0.05 vs. appropriate WT; † p < 0.05 vs. appropriate untreated.
(TIF)
