# Supplementary figures and images for: Characterization of hepatic macrophages and evaluation of inflammatory response in heme oxygenase-1 deficient mice exposed to scAAV9 vectors
Source: PLoS One. 2020 Oct 15;15(10):e0240691. doi: 10.1371/journal.pone.0240691 (PMC7561190; doi:10.1371/journal.pone.0240691)

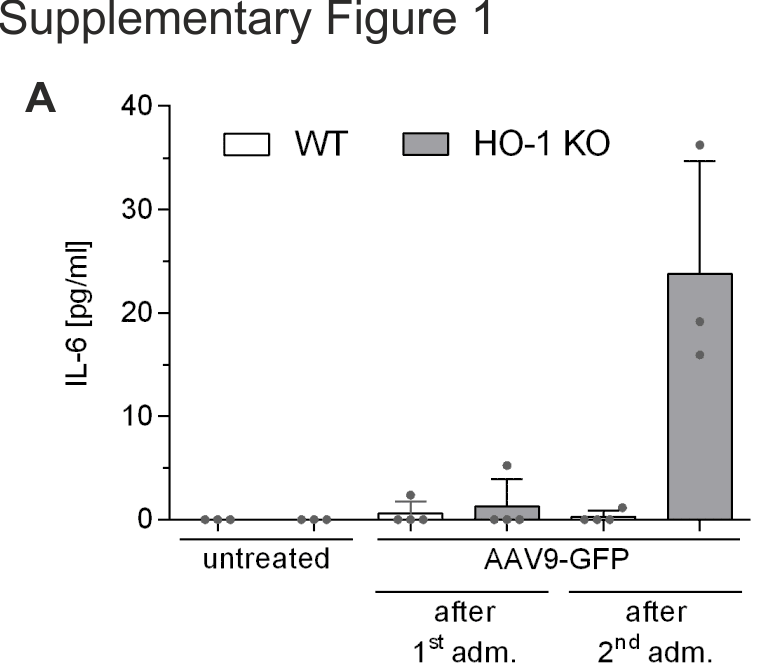

Supplement: S1 Fig — ELISA assessment of IL-6 in plasma collected from untreated mice, 3 days after 1st administration and 3 days after 2nd administration of scAAV9-GFP. (TIF) [file pone.0240691.s001.tif]

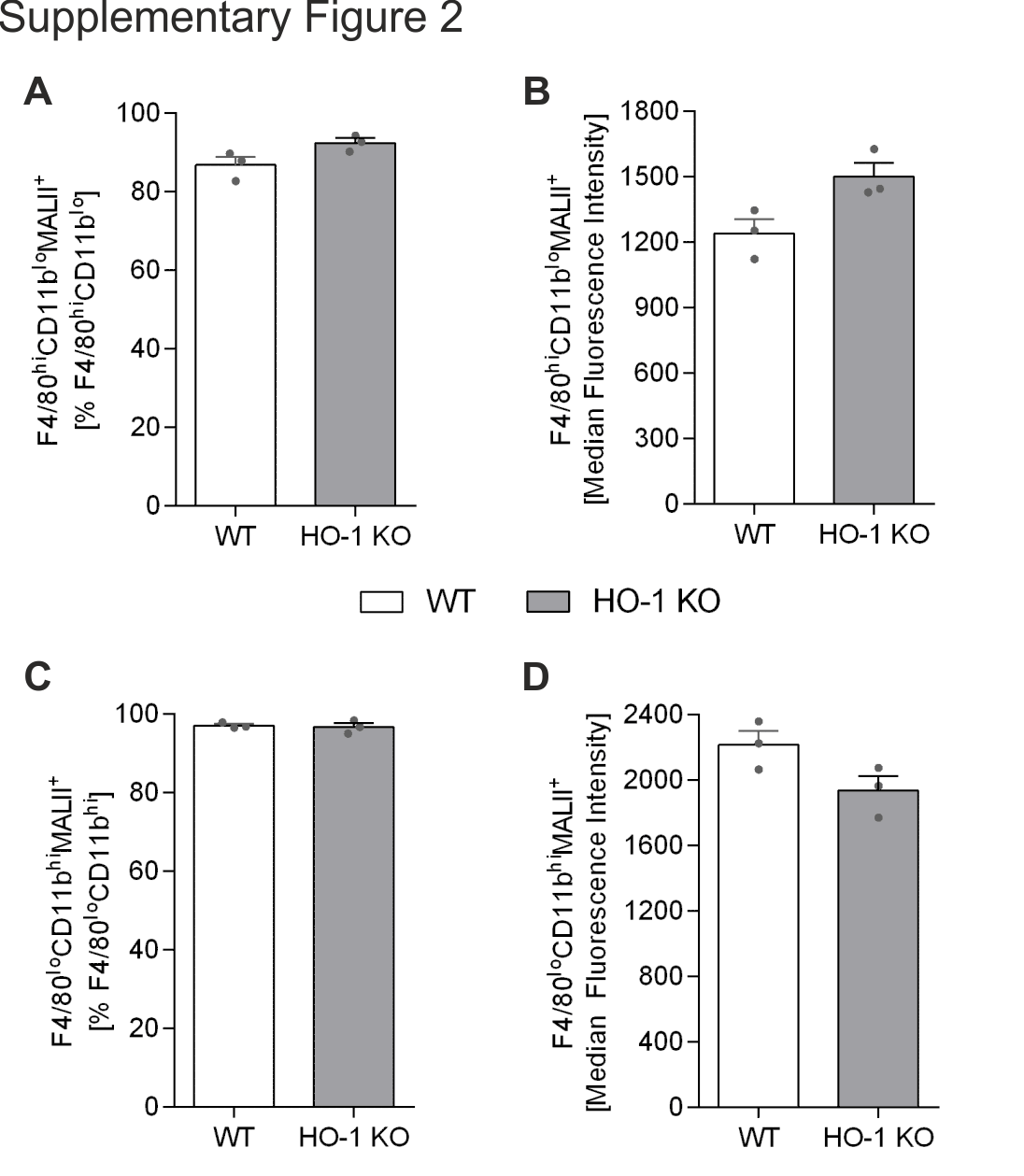

Supplement: S2 Fig — Maackia amurensis lectin-II (MAL-II) binds sialic acid attached to terminal galactose in α-2,3 linkage. (A) Percentage of F4/80highCD11blow cells labelled with MALII and (B) their median fluorescence intensity. (C) Percentage of F4/80lowCD11bhigh cells labelled with MALII and (D) their median fluorescence intensity. (TIF) [file pone.0240691.s002.tif]

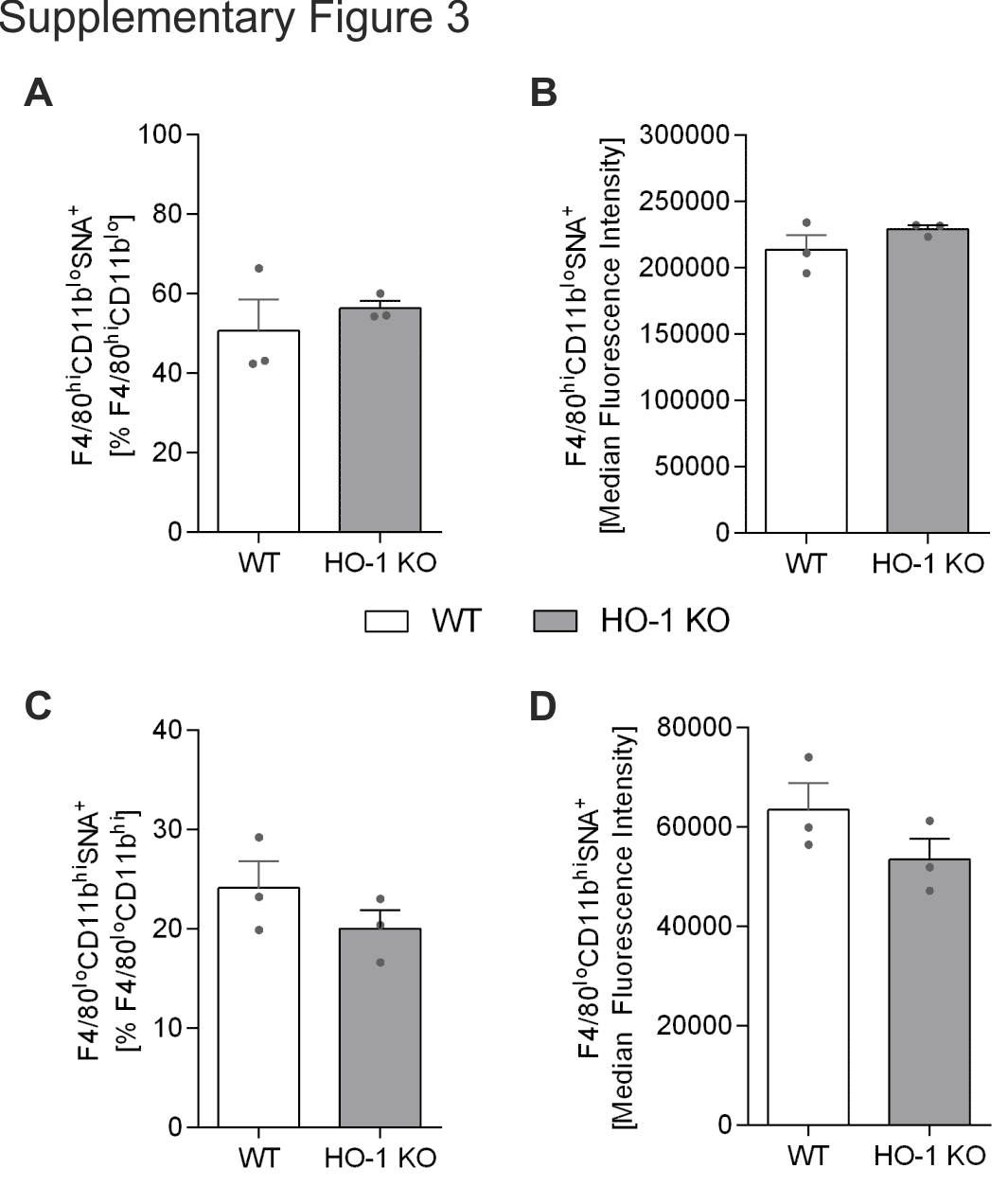

Supplement: S3 Fig — Sambucus nigra agglutinin (SNA) binds sialic acid attached to terminal galactose in α-2,6 linkage. (A) Percentage of F4/80highCD11blow cells labelled with SNA and (B) their median fluorescence intensity. (C) Percentage of F4/80lowCD11bhigh cells labelled with SNA and (D) their median fluorescence intensity. (TIF) [file pone.0240691.s003.tif]

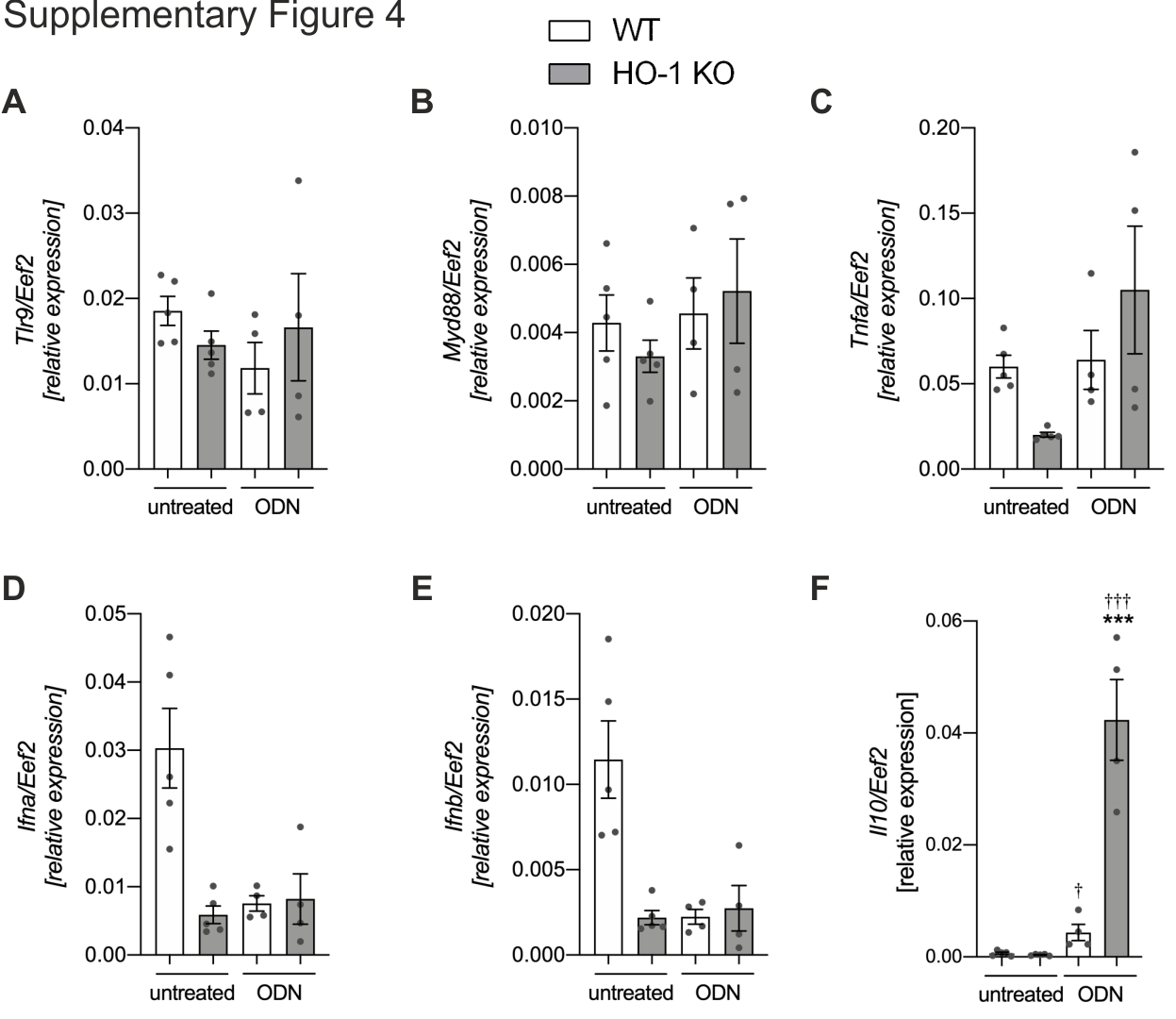

Supplement: S4 Fig — The qPCR for (A) Tlr9, (B) Myd88, (C) Tnfa, (D) Ifna, (E) Ifnb, and (F) Il10 at 24 h after in vitro stimulation of WT and HO-1 KO KCs with ODN 1585. *p < 0.05 vs. appropriate WT; † p < 0.05 vs. appropriate untreated. (TIF) [file pone.0240691.s004.tif]
